# Application of the Ridden Horse Pain Ethogram to Horses Competing at the Hickstead-Rotterdam Grand Prix Challenge and the British Dressage Grand Prix National Championship 2020 and Comparison with World Cup Grand Prix Competitions

**DOI:** 10.3390/ani11061820

**Published:** 2021-06-18

**Authors:** Sue Dyson, Danica Pollard

**Affiliations:** 1 The Cottage, Church Road, Market Weston, Diss IP22 2NX, UK; 2 The Rodhams, Rodham Road, Christchurch, Wisbech PE14 9NU, UK; drdee.pollard@gmail.com

**Keywords:** equine, lameness, performance, behaviour, head position, rein back, canter, piaffe, passage, pirouettes

## Abstract

**Simple Summary:**

The Ridden Horse Pain Ethogram (RHpE) comprises 24 behaviours, the majority of which are at least 10 times more likely to be seen in a horse with musculoskeletal discomfort than a non-lame horse. A RHpE score of eight or more indicates the presence of musculoskeletal pain. The most frequent RHpE score for 147 competitors at World Cup Grand Prix events from 2018 to 2020 was 3/24 (range 0–7), indicating that the majority of horses were working comfortably. The aim of the current study was to apply the RHpE to 38 competitors at the Hickstead-Rotterdam Grand Prix Challenge and 26 competitors at the British Dressage Grand Prix National Championship in 2020. The most frequent RHpE scores were 4 (range 0–8) and 6 (range 1–9), respectively. These scores were significantly higher than the World Cup competitors. This was associated with a higher prevalence of lameness and gait abnormalities in canter and more frequent errors in passage and piaffe, canter flying changes, canter pirouettes and rein back in the non-elite horses compared with the World Cup competitors. There was a negative correlation between the RHpE scores and the judges’ scores. Appropriate investigation and targeted management of horses with musculoskeletal discomfort may enhance both their performance and welfare.

**Abstract:**

The Ridden Horse Pain Ethogram (RHpE) comprising 24 behaviours was developed to facilitate the identification of musculoskeletal discomfort, with scores of ≥8/24 indicating the presence of pain. The median RHpE score for 147 competitors at World Cup Grand Prix events from 2018 to 2020 was three (interquartile range [IQR] 1–4; range 0–7). The aim of the current study was to apply the RHpE to 38 competitors at the Hickstead-Rotterdam Grand Prix Challenge and 26 competitors at the British Dressage Grand Prix National Championship in 2020. The median RHpE scores were four (IQR 3–6; range 0–8) and six (IQR 4–7; range 1–9), respectively, which were both higher (*p* = 0.0011 and *p* = 0.0000) than the World Cup competitors’ scores. Ears back ≥ 5 s (*p* = 0.005), intense stare ≥ 5 s (*p* = 0.000), repeated tail swishing (*p* = 0.000), hindlimb toe drag (*p* = 0.000), repeated tongue-out (*p* = 0.003) and crooked tail-carriage (*p* = 0.000) occurred more frequently. These were associated with a higher frequency of lameness, abnormalities of canter, and errors in rein-back, passage and piaffe, canter flying-changes and canter pirouettes compared with World Cup competitors. There was a moderate negative correlation between the dressage judges’ scores and the RHpE scores (Spearman’s rho −0.66, *p* = 0.0002) at the British Championship. Performance and welfare may be improved by recognition and appropriate treatment of underlying problems.

## 1. Introduction

The social licence to ride and to compete horses is coming under increasing focus [1,2,3,4,5]. The Ridden Horse Pain Ethogram (RHpE) was developed to facilitate the differentiation between horses with and without musculoskeletal discomfort [6]. It comprises 24 behaviours and a total RHpE score of ≥8/24 displayed in a period of approximately 10 min of ridden exercise is likely to reflect the presence of musculoskeletal pain. Significant reductions in the RHpE scores after the abolition of pain causing lameness by the use of diagnostic anaesthesia indicates a causal relationship between pain and these behaviours [7,8].

At five-star three-day events, a RHpE score of ≥7/24 during the warm-up for the dressage phase was associated with higher dressage penalties, an increased likelihood of being eliminated or retired in the cross-country phase and lower finish placings compared with horses that scored < 7/24 [9]. Scores of ≥7/24 were also associated with likely pain-related abnormalities of trot or canter. It was suggested that further investigation of elite event horses with RHpE scores of ≥7/24 may enable targeted treatment with the potential to improve both performance and welfare.

In order to compete in Grand Prix dressage competitions, horses must be at least eight years of age. The median RHpE score for 147 elite dressage competitors at the Fédération Equestre Internationale (FEI) Grand Prix World Cup (WC) qualifying competitions (*n* = 7) and finals (*n* = 2) was 3/24 (range 0–7) [10]. There was a moderate negative correlation between the judges’ scores and the RHpE scores.

There is an absence of published data relating to the prevalence of gait abnormalities in non-elite dressage horses. However, it is the first author’s impression, from many years of observation of horses in training and competitions and through a review of the results of clinical investigations and pre-purchase examinations, that the prevalence of pain-related gait abnormalities is higher in lower-level horses compared with the horses that qualified to compete in FEI WC competitions (unpublished data).

The purpose of this study was to apply the RHpE to a broader range of Grand Prix dressage horses than those elite horses that qualified to compete at FEI WC competitions and to compare the RHpE scores with performance. It was hypothesised that the median RHpE scores and range would be higher than those documented for horses competing in FEI WC competitions. Another objective was to document the frequency of the occurrence of specific movements which were not performed correctly according to the FEI guidelines [11].

## 2. Materials and Methods

### 2.1. Data Acquisition

The study was approved by the Ethics Review Panel of the Royal College of Veterinary Surgeons (2020–26). Video footage of horses competing at the Hickstead-Rotterdam Grand Prix Dressage (H-R) Challenge, September 2020 and the British Dressage Grand Prix National (BD) Championship, December 2020 was available via Horse and Country TV. For the H-R Challenge, horses performed outside on similar arena surfaces at both Hickstead, Sussex, UK and Rotterdam, The Netherlands. Invited participants at Hickstead represented Great Britain (17), Ireland (2) and Australia (1), whereas participants in Rotterdam represented The Netherlands (13), Belgium (3), Denmark (1) and Finland (1). All horses competing at the BD Championship, Hartpury University, Gloucestershire had qualified via preliminary competitions, according to the British Dressage Guidelines (Supplementary Materials S1) [12]. The competition took place in an indoor arena.

The video recordings at each venue were acquired in a standardised manner, so that each horse at a particular venue was viewed from similar perspectives for each movement of the FEI Grand Prix dressage test (Supplementary Materials S2) [11]. The horse occupied the majority of the screen throughout the video recordings. The duration of the test is approximately 6.5 min. The video recordings at each event were assessed live in chronological order, in real-time, and the RHpE (Supplementary Table S1) was applied by a single trained assessor (SD). Some horses repeatedly swished their tail throughout the test; others did so sporadically. It was noted, when possible, if the tail swishing coincided with the application of a spur cue. Additional free-hand comments were recorded concerning whether alterations in behaviour were provoked by specific movements (for example head behind vertical ≥10° for ≥10 s in passage and piaffe) and how movements were performed relative to the FEI guidelines (for example, number of one-time flying changes) [11]. Audio was switched off during the evaluation of the video recordings in order to avoid the introduction of bias based on the remarks made by the commentators.

There were no professional judges for the H-R Challenge, although scores were allocated via remote audience participation, but these were not analysed further. The dressage test at the BD Championship was judged by a panel of five professional judges, situated at different standardised locations around the 60 × 20 m arena. Each movement within the test was allocated a score (0–10; Supplementary Materials S2) according to FEI guidelines [11], independently by each judge. Some movements have a coefficient of two (for example, piaffe) and are thus marked out of 20. In addition, there is a final collective mark for the ‘rider’s position and seat and correctness and effect of the aids’, also with a coefficient of two. The final total score, out of a potential sum of 460, was the mean score of the five judges, expressed as a percentage. The final scores were recorded at the end of each competition. Age, breed and sex data for all horses were available from the FEI web site and were documented.

All horses had to wear a cavesson or crank cavesson noseband and double bridle. The noseband fit was checked by a steward at the termination of each test according to FEI guidelines, using an index finger at the side of the nasal bones [13]. All riders had to wear spurs and their appropriateness was also assessed by the steward.

The overall data for the RHpE scores and the individual behaviours exhibited by each horse from the H-R Challenge and the BD Championship were documented and compared. The correlation between the RHpE scores and the percentage scores were assessed for the BD Championship. Repeatability of observations was assessed for those horses that participated in both events. Data were compared with the results of the assessment, using a similar methodology, of the horses competing at FEI WC competitions [10].

On a separate occasion, at least one month after each competition, the video recordings were re-reviewed, without reference to the RHpE or judges’ scores. The movement ‘halt at C-immobility-rein back five steps-proceed immediately at collected trot’, was analysed according to the presence or absence of the following 13 features: head behind vertical ≥10° in halt, mouth open with separation of the teeth in halt, halt not square, halt not sustained, halt not at marker, head behind vertical ≥10° in rein back, mouth open in rein back, lack of diagonal steps in rein back, hindlimb(s) dragged in rein back, forelimb(s) dragged in rein back, rein back crooked, incorrect number of steps, other (for example, walked after rein back). The movement ‘extended walk’ was also assessed to determine if the position of the front of the horse’s head was behind the vertical ≥10° for > three steps. The video recordings could be stopped and replayed in real-time.

Gait abnormalities were also recorded as present or absent, including episodic forelimb lameness; hindlimb toe drag in trot; extended trot or passage; canter lacking a suspension phase; or a variable spatial and temporal separation of the hindlimbs in canter flying changes. Other abnormalities of passage, piaffe and canter pirouettes that did not comply with FEI Guidelines [11] were also recorded independently.

### 2.2. Data Analysis

Data were collated using Microsoft Excel (Office 365; Microsoft Corporation, Redmond, WA, USA). All coding and statistical analyses were conducted using Stata (IC v.13.0; StataCorp LP, College Station, TX, USA). Data distribution of continuous variables was checked for normality using a combination of visual assessment (histograms with overlaid normal and kernel density plots) and the Shapiro–Wilk test. Continuous and ordinal variables (horse age, dressage judges’ score, RHpE score and number of errors for the movement ‘halt-immobility-rein back five steps-collected trot’) were described as medians with a corresponding interquartile range (IQR) and range. Categorical variables (horse sex and the prevalence of behavioural and gait abnormalities) were described as proportions (%). The Spearman’s rank correlation coefficient was used to test if a correlation existed between the RHpE and the dressage judges’ scores for the BD Championships and to estimate the strength of the relationship. The absolute magnitude of the observed correlation coefficient was interpreted as: 0.00 to 0.10 (negligible correlation), 0.10 to 0.30 (weak correlation), 0.40 to 0.69 (moderate correlation), 0.70 to 0.89 (strong correlation) and 0.90 to 1.00 (very strong correlation) [14]. The differences among the competitions for the frequency of individual behaviours and the performances of specific movements were assessed using Chi-square or Fisher’s Exact Test, as appropriate. The Kruskal-Wallis and post-hoc Dunn’s tests were used to evaluate the differences in the RHpE scores. Significance was set at *p* < 0.05. *p*-values were not adjusted for multiple comparisons unless otherwise stated [15].

## 3. Results

### 3.1. Hickstead-Rotterdam Grand Prix Challenge

There were 38 horses, all Warmbloods, with a median age of 12 years (IQR 11–14; range 9–19). Geldings (*n* = 27, 71.1%) and stallions (*n* = 8, 21.1%) predominated, with a small proportion of mares (*n* = 3, 7.9%). The median RHpE score was 4/24 (IQR 3–6; range 0–8). The most frequent behaviours were mouth opening with separation of the teeth for ≥10 s (*n* = 28, 73.7%), head behind vertical ≥10° for ≥10 s (*n* = 25, 65.8%), an intense stare for ≥5 s (*n* = 21, 55.3%) and repeated tail swishing (*n* = 20, 52.6%).

Overall, 16 horses (42.1%) showed gait abnormalities in trot; one showed forelimb lameness in the left half-pass only and 15 exhibited hindlimb gait abnormalities (for example, toe drag in the extended trot and passage; lack of hindlimb impulsion). Gait abnormalities in canter (for example, lack of a suspension phase or a variable separation of the hindlimbs in flying changes) were observed in 13 horses (34.2%). The horse with a RHpE score of 8/24 had a persistent bilateral hindlimb toe drag in all trot work.

Abnormalities of gait in passage and/or piaffe were seen in 28 horses (73.7%). Flying changes were incorrect (missed changes, swinging excessively from side to side, croup high) in 16 horses (42.1%). Eleven horses (28.9%) placed the hindlimbs closely together temporally in canter pirouettes, slowed the rhythm or ‘jumped’ out of the movement. The front of the head was ≥10° behind vertical for > three steps in the extended walk in 16 horses (42.1%). The median score for errors in the movement ‘halt-immobility-rein back-collected trot’ was 2.5/13 (IQR 2–4; range 0–7), with the most frequent errors being rein back mouth open (*n* = 23, 63.9%), rein back head behind vertical ≥10° (*n* = 19, 52.8%) and halt not square (*n* = 12, 33.3%).

### 3.2. British Dressage Grand Prix National Championship

There were 26 horses, comprising 25 Warmbloods and one cob-cross, 19 (73.1%) geldings, two (7.7%) stallions and five (19.2%) mares, with a median age of 12.5 years (IQR 11–15; range 9–19). The median RHpE score was 6/24 (IQR 4–7; range 1–9). The most common behaviours were mouth opening with separation of the teeth for ≥10 s and head behind vertical ≥10° for ≥10 s (both *n* = 23, 88.5%), repeated tail swishing (*n* = 21, 80.8%), intense stare ≥ 5 s (*n* = 20, 76.9%) and ears back ≥ 5 s (*n* = 14, 53.9%).

Overall, gait abnormalities in trot were seen in 14 horses (53.8%), four with forelimb lameness seen in half pass (including two with hindlimb lameness) and 12 with hindlimb lameness (for example, toe drag in extended trot and passage; lack of hindlimb impulsion). Four horses (15.4%) showed gait abnormalities in canter. Three horses had a RHpE score of 8 or 9 and their gait abnormalities and incorrect performance of movements are summarised in Table 1.

Twenty-one horses (80.8%) had abnormal passage and/or piaffe, 12 horses (46.2%) performed flying changes poorly and eight horses (30.8%) displayed abnormal canter pirouettes. The front of the head was behind the vertical ≥10 s for > three steps in the extended walk in seven horses (26.9%). The median error score for the movement ‘halt-immobility-rein back-collected trot’ was 3/13 (IQR 2–4; range 0–7). The most frequent errors were rein back head behind vertical ≥10° (*n* = 18, 72.0%), rein back mouth open with separation of the teeth (*n* = 10, 40.0%) and halt head behind vertical ≥10° (*n* = 9, 36.0%).

**Figure 1:**
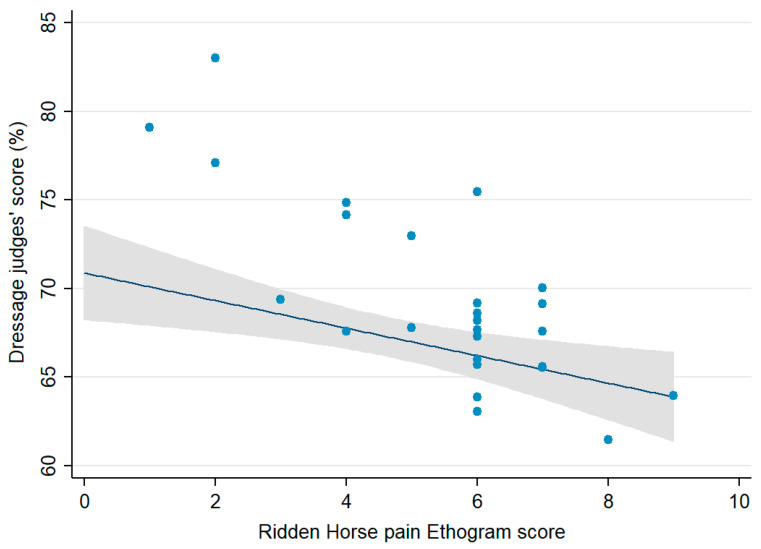
Comparison of the dressage judges’ percentage scores and the Ridden Horse Pain Ethogram scores (0–24) for horses competing at the British Dressage Grand Prix National Championship 2020 (*n* = 26), showing the individual scores, fitted line prediction and 95% confidence interval (CI). There was a moderate negative correlation, Spearman’s rho −0.6648, *p* = 0.0002.

The judges’ median score was 68.0% (IQR 65.7–73.0%; range 61.5%–83.0%). There was a moderate negative correlation between the dressage judges’ percentage scores and the RHpE scores (Spearman’s rho −0.66, *p =* 0.0002) (Figure 1).

### 3.3. Consistency of RHpE Scores in Horses Assessed at the Two Competitions

Six horses participated in both events and the RHpE scores for each horse were fairly consistent in the majority (7–6; 8–8; 6–6; 1–2; 6–6), with one notable exception (1–7). The latter horse showed major problems with all canter movements at the second competition (for example, missed flying changes, broke in canter pirouette). The consistency of the display of the ten most frequently observed behaviours is summarised in Table 2.

### 3.4. Comparison of Performance with Horses Competing at World Cup Competitions

Horses competing at the H-R Challenge and the BD Championship had a higher frequency of occurrence of gait abnormalities compared with those competing in WC competitions [10]. This is summarised in Table 3. Significant differences across the competitions were found for both trot (χ^2^ = 19.5, *p* < 0.001) and canter (χ^2^ = 22.3, *p* < 0.001).

There was a higher frequency of occurrence of gait abnormalities in passage and/or piaffe, flying changes and canter pirouettes in the current study compared with the horses competing in the WC competitions [10]. This is summarised in Table 4. Significant differences across the competitions were found for both flying changes (χ^2^ = 12.3, *p* = 0.002) and canter pirouettes (χ^2^ = 9.4, *p* = 0.009), but not for passage and/or piaffe (χ^2^ = 4.7, *p* = 0.108).

**Figure 2:**
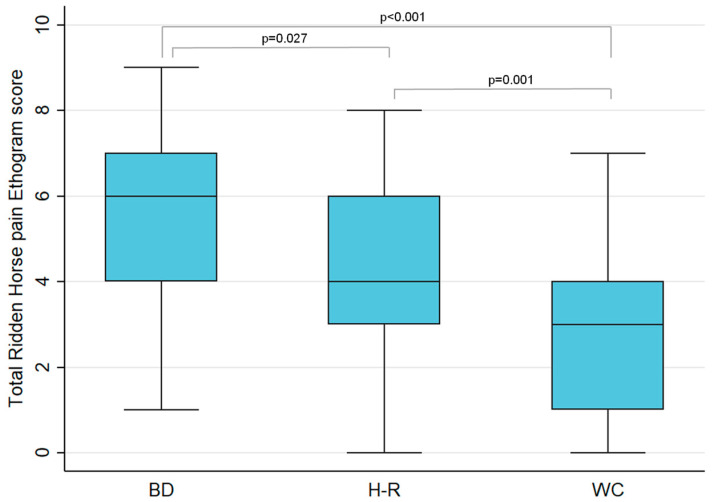
Total Ridden Horse Pain Ethogram scores (out of 24) for competitors at the British Dressage Grand Prix National Championship (BDC) (*n* = 26), the Hickstead-Rotterdam Challenge (HRC) (*n* = 38) and the World Cup Western League qualifying competitions and the finals (*n* = 147) (WC) [10]. There were significant differences between each of the competitions as determined by the Kruskal-Wallis and post-hoc Dunn’s test. Boxes represent the medians and interquartile ranges while whiskers represent the range.

There were significant differences in the median RHpE scores among all three competitions. The median RHpE scores were significantly higher in the horses competing at the BD Championship compared with the H-R Challenge (*p* = 0.0267), and WC competitions (*p* = 0.0000). The H-R Challenge median RHpE score was also higher compared with WC competitions (*p* = 0.0011) [10] (Table 5, Figure 2).

The frequency of occurrence for the majority of RHpE behaviours was higher for the BD Championship and H-R Challenge compared with WC competitions [10] (Table 6). The differences were significant for ears back ≥ 5 s (*p* = 0.005), intense stare ≥ 5 s (*p* = 0.000), tongue out repeatedly (*p* = 0.003), tail crooked (*p* = 0.000), tail swishing repeatedly (*p* = 0.000), bilateral hindlimb toe drag (*p* = 0.000) and reluctance to go forwards (*p* = 0.024).

There was a significantly higher frequency of occurrence of three errors (mouth open in rein back *p* = 0.004, lack of diagonal steps *p* = 0.028, and crooked in rein back *p* = 0.027) in the movement ‘halt-immobility-rein back-collected trot’ in the horses competing in the H-R Challenge and the BD Championship compared with the WC competitions (Table 7), although the median total error scores were similar (3 and 2/13, respectively).

## 4. Discussion

In accordance with the hypothesis, the horses in the current study had higher median RHpE scores compared with the horses in WC competitions, despite 10 horses being included in both data sets. The most frequently observed behaviours generally had a higher frequency of occurrence in the horses in the current study compared with the horses competing in WC competitions, and a larger number of behaviours occurred in >10% of the horses. This was associated with a higher frequency of both gait abnormalities in trot and canter and errors in rein back, passage and piaffe, canter flying changes and canter pirouettes. It seems likely that a proportion of the lower-level Grand Prix horses are experiencing musculoskeletal discomfort and with appropriate investigation and management both their welfare and performance could be enhanced.

In particular, there was a higher frequency of occurrence of an intense stare for ≥5 s, repeated tail swishing, ears behind vertical for ≥5 s, repeated hindlimb toe drag, repeated exposure of the tongue, reluctance to go forwards and a crooked tail in the competitors in the current study compared with those competing in the WC competitions [10]. An association between crooked tail carriage and hindlimb lameness, epaxial muscle tension and sacroiliac joint region pain has previously been documented [16]. Repeated tail swishing was observed in the majority of competitors and is specifically mentioned in the FEI Dressage Rules as a ‘sign of nervousness, tension or resistance on the part of the Horse and must be taken into account by the Judges in their marks for every movement concerned, as well as in the collective mark’ [11]. Repeated exposure of the tongue was observed less frequently but this is also regarded as a serious fault which should be penalised appropriately. In a previous study, ears behind vertical for ≥5 s and bilateral hindlimb toe drag were seen with a 34% and 32% higher prevalence, respectively in lame versus non-lame horses [17]. There was a stronger negative correlation between the RHpE scores and the judges’ scores for the horses in the current study (Spearman’s rho −0.66, *p* = 0.0002) than for the elite WC horses (Spearman rho −0.40, *p* < 0.001) [10], which is likely to reflect the higher frequency of gait abnormalities in the non-elite horses influencing the quality of their performances.

However, these observations also need to be put into perspective. In a convenience sample of United Kingdom sports and leisure horses, in full work and presumed by their riders to be working comfortably, 25/148 horses were dressage horses competing from Novice to Advanced Medium level [18]. The median age of the dressage horses was 9 years (range 6–26), younger than the horses in the current study. These horses had a substantially higher median RHpE score of 9 [IQR 5–10] compared with the current study, which was comprised of horses competing at a considerably higher level. There was a positive association between the RHpE score and lameness [18]. Grand Prix dressage horses have to be at least eight years of age and many are considerably older, perhaps reflecting a ‘survival of the fittest’, especially among the elite horses. A ‘healthy horse effect’, similar to the healthy worker effect seen in humans [19], has previously been recorded for equine foot pain [20], whereby older animals that are still in work are less predisposed to injury.

When comparing the results of the current study with those of elite five-star three-day event horses warming-up for the dressage phase on a grass surface, the most frequent observations were similar including front of the head behind a vertical position ≥10° for ≥10 s, repeated head tilt, mouth open with separation of the teeth for ≥10 s, an intense stare for ≥10 s and repeated tail swishing [9]. However, the frequency of occurrence of mouth open with separation of the teeth for ≥10 s, an intense stare for ≥5 s and repeated tail swishing was considerably higher for the dressage horses (Supplementary Table S2). Moreover, several other behaviours, including ears back behind vertical ≥5 s, spontaneous changes of gait, bilateral hindlimb toe drag or stumbling, and repeated exposure of the tongue occurred with a much higher frequency in the dressage horses compared with the event horses. These observations, combined with higher median RHpE scores for the dressage horses, are suggestive of a higher level of discomfort among the dressage horses compared with the event horses.

However, it should be borne in mind that the event horses were assessed during the preparation for a dressage test on a grass surface, whereas the dressage horses were assessed while performing the competition test on an arena surface. The degree of collection [21,22,23] and the complexity of the movements [24,25,26,27] are higher for the Grand Prix dressage horses compared with the event horses. Advanced diagonal placement [21], or placing the load on a single hindlimb in trot, is relatively unusual in event horses, but is more common in elite dressage horses. The event horses have a much more varied training and may be less predisposed to repetitive strain injuries compared with dressage horses [28]. In addition, it is not compulsory for event horses to use a double bridle and the majority used snaffle bits, with a wider range of noseband types with the potential to restrict the mouth opening compared with the dressage horses. The substantially higher occurrences of mouth opening and the tongue being out in the dressage horses is suggestive of a possible causal association between these behaviours and the bit type which merits further investigation.

As previously documented for elite Grand Prix competitors [10], in the current study there were rider or training errors that resulted in a potentially avoidable loss of marks for inaccuracies, for example halt not being at the marker and an incorrect number of rein back steps. Although the high frequency of occurrence of head behind vertical ≥10° for ≥10 s in any movement may be compounded by the presence of musculoskeletal discomfort, it seems likely that this may also be in part a reflection of modern-day training. The observation of head behind vertical increased in frequency among elite Grand Prix horses between 1992 and 2008 [29]. It is clearly not being heavily penalised by judges. This head and neck posture may have adverse consequences for the optimal development of the cervical muscles, the epaxial and hypaxial muscles of the thoracolumbosacral regions, the muscles of the thoracic sling, the pelvic and hindlimb muscles and the abdominal ‘core’ muscles and for the establishment of correct movement patterns of the thoracolumbosacral region, forelimbs and hindlimbs [30,31,32], factors which may have the potential to predispose to injury. The high frequency of gait abnormalities in trot and canter in the current study and the inability to perform correctly many of the movements requiring increased collection may reflect the consequences of inadequacies in basic training. The failure to recognise pain-related gait abnormalities has welfare implications and may jeopardise longevity of performance. Improvements in basic training, by establishing more correct movement patterns, could potentially reduce the risks of repetitive strain injuries.

It is acknowledged that some aspects of behaviour such as repeated tail swishing may be a manifestation of conflict behaviour or stress [33,34,35]. It should be noted that overall there was a higher frequency of occurrence of tail swishing than recorded in the RHpE data, because if tail swishing consistently coincided with the application of a leg or spur cue, it was recorded but not included as a positive finding in the RHpE. The potential causes of mouth opening and other factors influencing the head and neck position in Grand Prix dressage horses are discussed further elsewhere [10]. However, overall, combining the RHpE data with the abnormalities of gait which were observed in the current study, it is concluded that high RHpE scores were likely to reflect discomfort. When considering the relationship between the RHpE score and the judges’ scores it is important to recognise that a horse which has undergone correct training and has no pain-related gait abnormalities can have a low RHpE score, but through lack of experience and making mistakes, or through having lower quality natural paces, the horse will ultimately finish with a lower percentage judges’ score than a more experienced pain-free horse, or a pain-free horse with naturally superior quality of paces. A correctly trained horse that is pain free should be able to perform movements according to FEI Guidelines, but may make errors due to a lack of musculoskeletal strength and coordination, however these should not be accompanied by signs consistent with pain.

The study had some limitations. The video recordings were acquired in a standardised way at each venue, but varied slightly among venues, which meant that some movements were observed from different perspectives. The competitors could not be anonymised. There were 10 elite horses that competed in both a WC competition and in a competition in the current study; this had the potential to reduce the differences between the data sets, but nonetheless major differences were observed without excluding these horses from the analyses. There was a smaller number of non-elite competitors compared with the WC competitors, likely limiting the statistical power of the study. There was only a single experienced assessor, however a high level of repeatability of application of the RHpE has previously been shown for trained assessors [6,10,36]. In a previous study we also showed no significant difference in real-time scores and video-based scores for an experienced assessor [36]. Moreover, having the same assessor for the WC competitions and the two competitions in the current study provided consistency for a comparison of the results. The same person evaluated the gaits and the correctness of movements according to FEI guidelines, with the potential for bias; however, this was performed without the knowledge of the judges’ scores and at least one month after assigning the RHpE scores. Moreover, all statistical data analysis was performed independently.

## 5. Conclusions

The median RHpE scores for non-elite Grand Prix dressage horses were higher than for elite horses, in association with a higher frequency of occurrence of both low-grade lameness and abnormalities of canter and also the incorrect performance of many movements requiring collection. An appropriate investigation into those horses with evidence of musculoskeletal discomfort would permit targeted management strategies and enhance both welfare and performance.

## Figures and Tables

**Table 1:** Summary of gait abnormalities of three horses that had a Ridden Horse Pain Ethogram (RHpE) score of 8 or 9/24 when competing at the British Dressage Grand Prix National Championship.

Horse Number	RHpE Score	Gait Abnormalities
1	8	Bilateral hindlimb toe drag in passage; canter lacked suspension phase; canter crooked; reduced range of motion of lumbosacral region; stopped lifting limbs in piaffe—‘stuck’
2	8	Bilateral hindlimb toe drag in passage and extended trot; hindlimbs crossed in passage; inadequate engagement of hindlimbs; reduced range of motion of lumbosacral region; close temporal placement of hindlimbs in canter pirouettes and loss of rhythm
3	9	Broke to walk in second half-pass; unilateral hindlimb toe drag in passage; forelimbs crossed in passage; low hindlimb steps in piaffe and irregular rhythm; slowed rhythm in canter pirouettes

**Table 2:** Summary of the frequency and consistency of the ten most commonly observed behaviours of the Ridden Horse Pain Ethogram in six horses assessed at two Grand Prix dressage competitions.

Behaviour	Horses Showing Behaviour at Least Once	Horses Showing Inconsistency	Horses Showing Consistency
Tail swishing large movements: repeatedly up and down/side to side/circular; repeatedly during transitions	6	1	5
Mouth opening ± shutting repeatedly with separation of teeth, for ≥10 s	5	0	5
Ears rotated back behind vertical (both or one only) for ≥5 s; repeatedly lay flat	5	2	3
Intense stare (glazed expression, ‘zoned out’) for ≥5 s	5	2	3
Head behind vertical (≥10°) for ≥10 s	4	2	2
Tail clamped tightly to middle or held to one side	4	4	0
Stumbles or trips more than once; repeated bilateral hindlimb toe drag	3	0	3
Head tilted or tilting repeatedly	2	1	1
Sclera exposed repeatedly	2	2	0
Spontaneous changes of gait (e.g., breaks from canter to trot or trot to canter)	2	2	0

**Table 3:** Comparison of the frequency of occurrence (percentage, %) of gait abnormalities in trot and canter at the World Cup Grand Prix Western League qualifying competitions (*n* = 7) and the finals (*n* = 2) 2018–2020 [10], the 2020 Hickstead-Rotterdam (H-R) Grand Prix Challenge and the 2020 British Dressage (BD) Grand Prix National Championship.

Competition	Number of Competitors	Number of Horses with Trot Abnormalities (%)	Number of Horses with Canter Abnormalities (%)
World Cup	147	27 (18.4)	9 (6.1)
H-R Challenge	38	16 (42.1)	13 (34.2)
BD Championship	26	14 (53.8)	4 (15.4)

**Table 4:** Comparison of the frequency of occurrence (percentages, %) of gait abnormalities in passage and/or piaffe, flying changes and canter pirouettes at the World Cup Grand Prix Western League qualifying competitions (*n* = 7) and the finals (*n* = 2) 2018–2020 [10], the 2020 Hickstead-Rotterdam Grand Prix (H-R) Challenge and the 2020 British Dressage Grand Prix (BD) National Championship.

Competition	Number of Competitors	Number of Horses with Passage and/or Piaffe Abnormalities (%)	Number of of Horses with Flying Change Abnormalities (%)	Number of of Horses with Canter Pirouette Abnormalities (%)
World Cup	147	91 (61.9)	30 (20.4)	18 (12.2)
H-R Challenge	38	28 (73.7)	16 (42.1)	11 (28.9)
BD Championship	26	21 (80.8)	12 (46.2)	8 (30.8)

**Table 5:** Comparison of Ridden Horse Pain Ethogram (RHpE) scores (0–24) at the World Cup Grand Prix Western League qualifying competitions (*n* = 7) and the finals (*n* = 2) 2018–2020 [10], the 2020 Hickstead-Rotterdam Grand Prix (H-R) Challenge and the 2020 British Dressage Grand Prix (BD) National Championship. Judges’ percentage (%) scores are also compared between the World Cup competitions and the BD Championship. IQR = interquartile range.

	Competition	Number of Competitors	Median	IQR	Range
RHpE score	World Cup	147	3	1,4	0,7
	H-R Challenge	38	4	3,6	0,8
	BD Championship	26	6	4,7	1,9
Judges’ score %	World Cup	147	72.1	68.4–75.3	58.3–83.6
	BD Championship	26	68.0	65.7–73.0	61.5–83.0

**Table 6:** Summary of the frequency of occurrence (percentage) for each of the behaviours (*n* = 24) of the Ridden Horse Pain Ethogram for all venues combined for competitors (*n* = 147) at the World Cup Grand Prix Dressage Western League qualifying competitions (*n* = 7) and the finals (*n* = 2) [10], the Hickstead-Rotterdam Grand Prix Challenge (*n* = 38) and the British Dressage Grand Prix National Championship (*n* = 26). Significant differences are highlighted in bold.

Behaviour	World Cup (%)	Hickstead-Rotterdam (%)	British Dressage (%)	*p*-Value *
Mouth open with separation of the teeth for ≥10 s	68.0	73.7	88.5	0.084
Front of head behind vertical ≥10° for ≥10 s	66.7	65.8	88.5	0.067
**Intense stare ≥ 5 s**	**29.9**	**55.3**	**76.9**	**<0.001**
**Repeated tail swishing not in synchrony with spur aids**	**28.6**	**52.6**	**80.8**	**<0.001**
**Ears back behind vertical ≥5 s**	**24.5**	**39.5**	**53.9**	**0.005**
Repeated head tilt	20.4	10.5	30.8	0.137
Spontaneous change of gait	8.8	15.8	19.2	0.182
**Repeated stumbling or bilateral hindlimb toe drag**	**6.8**	**26.3**	**34.6**	**<0.001**
Repeated exposure of the sclera	6.8	7.9	11.5	0.551
**Repeated exposure of the tongue**	**4.8**	**18.4**	**19.2**	**0.003**
Head moved from side to side	2.0	5.3	3.9	0.313
Spontaneous change of direction; spooking	2.0	2.6	0	1.000
Head movement up and down, not in synchrony with the trot rhythm	1.4	5.3	0	0.222
Bucking	0.7	2.6	3.9	0.20
Rearing	0.7	5.3	0	0.325
**Reluctance to go forwards**	**0.7**	**7.9**	**3.9**	**0.024**
Crooked, on 3 tracks	0.7	0	7.7	0.058
**Crooked tail, held to one side**	**0.7**	**13.2**	**23.1**	**<0.001**
Eyes partially closed 2–5 s; repeated blinking	0.7	0	0	1.000
Gait too slow	0	0	0	-
Rushed gait	0	0	0	-
Repeated incorrect strike off in canter	0	0	0	-
Bit pulled through to one side	0	0	0	-
Head in front of vertical ≥ 30° ≥ 10 s	0	0	0	-

* Chi-square/Fisher’s exact test *p*-value.

**Table 7:** Summary of the frequency of occurrence of errors in the movement ‘halt at C, immobility, rein back five steps, proceed in collected trot’ for all venues combined (146 competitors) for the World Cup Grand Prix Western League qualifying competitions (*n* = 7) and the finals (*n* = 2) (median score was 2/13 [interquartile range, IQR 1–3; range 0–8]) [10] and the combined results for the Hickstead-Rotterdam Grand Prix Challenge and the British Dressage Grand Prix National Championship (*n* = 61; data were missing for 3 competitors) (median score 3/13 [IQR 2–4; range 0–7]). Significant differences are highlighted in bold.

	World Cup		Hickstead-Rotterdam and British Dressage Championship		
Error	Number	Percentage	Number	Percentage	*p*-Value ^+^
Halt head behind vertical	26	17.8	18	30.0	0.061
Halt mouth open with separation of the teeth	13	8.9	9	14.8	0.213
Halt not square	52	35.6	19	31.2	0.537
Halt not sustained	14	9.6	6	9.8	0.956
Halt not at marker	25	17.1	15	24.6	0.215
Rein back head behind vertical ≥10°	92	63.0	37	60.7	0.750
**Rein back mouth open with separation of the teeth**	48	**32.9**	33	**54.0**	**0.004**
**Rein back lack of diagonal steps**	18	**12.3**	15	**24.6**	**0.028**
Rein back, hindlimb(s) dragged	6	4.1	3	4.9	0.795
Rein back, forelimb(s) dragged	13	8.9	6	9.8	0.832
**Rein back crooked**	8	**5.5**	9	**14.8**	**0.027**
Rein back incorrect number of steps	34	23.3	13	21.3	0.757
Other *	10	6.7	1	1.6	-

* Other = stepped back with one hindlimb after halting; raised head before or during rein back; variable rhythm of steps in rein back; walked between rein back and collected trot; jumped into collected trot; head in front of vertical in transition to collected trot; mouth open with separation of teeth in collected trot; ^+^ Chi-square/Fisher’s exact test *p*-value.

## Data Availability

Anonymised data are available from the corresponding author on reasonable request. The data are not publicly available in order to protect the confidentiality of participants.

## Supplementary Materials

The following are available online at https://www.mdpi.com/article/10.3390/ani11061820/s1, Supplementary Materials S1: British Dressage Qualifications for the Grand Prix National Championships, Supplementary Materials S2: The Fédération Equestre Internationale Grand Prix Dressage test, Table S1: Summary of the Ridden Horse Pain Ethogram (adapted from Dyson et al. 2018) [5], Table S2: Comparison of the frequency of occurrence (percentage) of the 24 behaviours of the Ridden Horse Pain Ethogram among horses warming-up for the dressage phase of 5* three-day events (TDE) (*n* = 137) [9] and Grand Prix dressage horses in competition in the Hickstead-Rotterdam Challenge (*n* = 38) and the British Dressage Grand Prix National Championship (*n* = 26).
