# Supplementary material for: Application of the Ridden Horse Pain Ethogram to Horses Competing at the Hickstead-Rotterdam Grand Prix Challenge and the British Dressage Grand Prix National Championship 2020 and Comparison with World Cup Grand Prix Competitions
Source: Animals (Basel). 2021 Jun 18;11(6):1820. doi: 10.3390/ani11061820 (PMC8235099; doi:10.3390/ani11061820)
Supplement: Supplementary file 1 [file animals-11-01820-s001.zip › animals-1211651-supplementary.pdf]

Supplementary items

Supplementary information 1: British Dressage Qualifications for the Grand Prix National Championships

Supplementary information 2: The Fédération Equestre Internationale Grand Prix Dressage test.

Supplementary information 3: Table S1. Summary of the Ridden Horse Pain Ethogram (adapted from Dyson *et al.* 2018) [5].

Supplementary information 4: Table S2. Comparison of the frequency of occurrence (percentage) of the 24 behaviours of the Ridden Horse Pain Ethogram among horses warming-up for the dressage phase of 5* three-day events (TDE) (n=137) [9] and Grand Prix dressage horses in competition in the Hickstead-Rotterdam Challenge (n=38) and the British Dressage Grand Prix National Championship (n=26).

Supplementary information 1

British Dressage Qualifications for the Grand Prix National Championships

Places for the Grand Prix Championships will be allocated as follows:

- Automatic qualification for the selected combinations for that year's Senior European or World Championships or Olympic Games
- Automatic qualification for the winners of each Premier League Grand Prix Gold class (one per Premier League) (excluding Grand Prix Special or Freestyle) scoring 65% or above.
- Qualification for the three combinations who have achieved the highest scores above 65% in UK based or overseas CDI Grand Prix classes (excluding under 25 Grand Prix, Grand Prix Special or Freestyle).
- Qualification for the three horses who achieve the highest average score above 65% from three Premier League or High Profile Show Grand Prix and under 25 Grand Prix classes, excluding Grand Prix Special or Freestyle. At least one score must come from a Premier League.

If the places are not filled by the above method, additional places will be filled by taking the next highest score from either the CDI list or the list of the highest average scores from three Premier League or High Profile Show Grand Prix classes.

Supplementary information 2.

The Fédération Equestre Internationale Grand Prix Dressage test.

	Markers at which movement performed	Instructions	Score	Coefficient	Directions to judges
1	A<br>X<br>XC	Enter in collected canter<br>Halt - immobility - salute<br>Proceed in collected trot<br>Collected trot	10		Quality of paces, halt, and transitions. Straightness. Contact and poll.
2	C<br>HXF<br>FAK	Track to the left<br>Extended trot<br>Collected trot	10		Regularity, elasticity, balance, of hindquarters, overtrack. Lengthening of frame. Both transitions.
3	KB	Half-pass to the right	10	2	Regularity and quality of trot, uniform bend, collection, balance, fluency, crossing of legs.
4	BH<br>HC	Half-pass to the left<br>Collected trot	10	2	Regularity and quality of trot, uniform bend, collection, balance, fluency, crossing of legs.
5	C	Halt - immobility<br>Rein back 5 steps and immediately proceed in collected trot	10		Quality of halt and transitions. Throughness, fluency, straightness. Accuracy in number of diagonal steps.
6	MV	Extended trot	10		Regularity, elasticity, balance, energy of hindquarters, overtrack. Lengthening of frame. Transition to extended trot.
7	VKD	Passage	10		Regularity, cadence, collection, self-carriage, balance, activity, elasticity of back and steps. Transition to passage.
8	D	Piaffe 12 to 15 steps	10	2	Regularity, taking weight, self-carriage, activity, elasticity of back and steps. Specific number of diagonal steps.
9	D	Transitions passage - piaffe - passage	10		Maintenance of rhythm, collection, self-carriage, balance, fluency, straightness. Precise execution.
10	DFP	Passage	10		Regularity, cadence, collection, self-carriage, balance, activity, elasticity of back and steps.
11	PH	Extended walk	10	2	Regularity, suppleness of back, activity, overtrack, freedom of shoulder, stretching to the bit. Transition into walk.
12	HCM	Collected walk	10	2	Regularity, suppleness of back, activity, shortening and heightening of steps, self-carriage.
13		Proceed in passage	10		Fluency, promptness,

		Transition collected walk - passage			self-carriage, balance, straightness.
14	MRI	Passage	10		Regularity, cadence, collection, self-carriage, balance, activity, elasticity of back and steps.
15	I	Piaffe 12 to 15 steps	10	2	Regularity, taking weight, self-carriage, activity, elasticity of back and steps. Specific number of diagonal steps.
16	I	Transitions passage - piaffe - passage	10		Maintenance of rhythm, collection, self-carriage, balance, fluency, straightness.<br>Precise execution.
17	ISE	Passage	10		Regularity, cadence, collection, self-carriage, balance, activity, elasticity of back and steps.
18	E<br>EKAF	Proceed in collected canter left<br>Collected canter	10		Precise execution and fluency of transition.<br>Quality of canter.
19	FXH<br>HCM	On the diagonal 9 flying changes of leg every 2nd stride<br>Collected canter	10		Correctness, balance, fluency, uphill tendency, straightness. Quality of canter before and after.
20	MXK	Extended canter	10		Quality of canter, impulsion, lengthening of strides and frame. Balance, uphill tendency, straightness.
21	K<br>KA	Collected canter and flying change of leg<br>Collected canter	10		Quality of flying change on diagonal.<br>Precise, smooth execution of transition.
22	A<br>Between D & G<br>G<br>C	Down the centre line<br>5 half-passes to either side of centre line with flying change of leg at each change of direction, the first half-pass to the left and the last to the left of 3 strides, the others of 6 strides<br>Flying change of leg<br>Track to the right	10	2	Quality of canter.<br>Uniform bend, collection, balance, fluency from side to side.<br>Symmetrical execution.<br>Quality of flying changes.
23	MXK<br>KA	On the diagonal 15 flying changes of leg every stride<br>Collected canter	10	2	Correctness, balance, fluency, uphill tendency, straightness. Quality of canter before and after.

24	A<br>L	Down the centre line<br>Pirouette to the left	10	2	Collection, self-carriage, balance, size, flexion and bend. Correct number of strides (6-8). Quality of canter before and after.
25	X	Flying change of leg	10		Correctness, balance, fluency, uphill tendency, straightness.<br>Quality of canter before and after.
26	I<br><br>C<br>CM	Pirouette to the right<br>Track to the right<br>Collected canter	10	2	Collection, self-carriage, balance, size, flexion and bend. Correct number of strides (6-8). Quality of canter before and after.
27	M<br>MR	Collected trot<br>Collected trot	10		Fluency; precise, smooth execution of transition. Collection.
28	RK<br>K<br>KA	Extended trot<br>Collected trot<br>Collected trot	10		Regularity, elasticity, balance, energy of hindquarters, overtrack. Lengthening of frame. Both transitions. Collection.
29	A<br>DX	Down the centre line<br>Passage	10		Regularity, cadence, collection, self-carriage, balance, activity, elasticity of back and steps. Transition to passage.
30	X	Piaffe 12 to 15 steps	10	2	Regularity, taking weight, self-carriage, activity, elasticity of back and steps. Specific number of diagonal steps.
31	X	Transitions passage - piaffe - passage	10		Maintenance of rhythm, collection, self-carriage, balance, fluency, straightness.<br>Precise execution.
32	XG	Passage	10		Regularity, cadence, collection, self-carriage, balance, activity, elasticity of back and steps.
33	G	Halt - immobility - salute	10		Quality of halt and transition. Straightness. Contact and poll.

Supplementary information 3

Table S1. Summary of the Ridden Horse Pain Ethogram (adapted from Dyson *et al.* 2018) [5].

Definitions of the 24 behaviours

1. Repeated changes of head position (up/down), not in rhythm with the trot
2. Head tilted or tilting repeatedly

3. Head in front of vertical (≥30^0) for ≥10s
4. Head behind vertical (≥10^0) for ≥10s
5. Head position changes regularly, tossed or twisted from side to side, corrected constantly
6. Ears rotated back behind vertical (both or one only) for ≥5s; repeatedly lay flat
7. Eye lids closed or half closed for 2-5 s; frequent blinking
8. Sclera exposed repeatedly
9. Intense stare (glazed expression, 'zoned out') for ≥5s
10. Mouth opening ± shutting repeatedly with separation of teeth, for ≥10 s
11. Tongue exposed, protruding or hanging out, and/or moving in and out repeatedly
12. Bit pulled through the mouth on one side (left or right), repeatedly
13. Tail clamped tightly to middle or held to one side
14. Tail swishing large movements: repeatedly up and down/side to side/ circular; repeatedly during transitions
15. A rushed gait (frequency of trot steps > 40/15s); irregular rhythm in trot or canter; repeated changes of speed in trot or canter
16. Gait too slow (frequency of trot steps <35/15s); passage-like trot
17. Hindlimbs do not follow tracks of forelimbs but repeatedly deviated to left or right; on 3 tracks in trot or canter
18. Canter repeated leg changes change of leg in front and / or behind; repeated strike off wrong leg; disunited
19. Spontaneous changes of gait (e.g., breaks from canter to trot or trot to canter)
20. Stumbles or trips more than once; repeated bilateral hindlimb toe drag
21. Sudden change of direction, against rider direction; spooking
22. Reluctance to move forwards (has to be kicked ± verbal encouragement), stops spontaneously
23. Rearing (both forelimbs off the ground)
24. Bucking or kicking backwards (one or both hindlimbs)

Reprinted from J. Vet. Behav.: Clin. Appl. Res., 23, Dyson, S., Berger, J., Ellis, A., Mullard, J., Development of an ethogram for a pain scoring system in ridden horses and its application to determine the presence of musculoskeletal pain, Page 53, Copyright (2018), with permission from Elsevier.

Supplementary information 4

Table S2. Comparison of the frequency of occurrence (percentage) of the 24 behaviours of the Ridden Horse Pain Ethogram among horses warming-up for the dressage phase of 5* three-day events (TDE) (n = 137) [9] and Grand Prix dressage horses in competition in the Hickstead-Rotterdam Challenge (n = 38) and the British Dressage Grand Prix National Championship (n = 26). Major differences are highlighted in bold.

Behaviour	TDE (%)	Hickstead-Rotterdam (%)	British Dressage (%)
**Mouth open with separation of the teeth for ≥10s**	**44.0**	**73.7**	**88.5**
Front of head behind vertical ≥10^0 for ≥10s	64.0	65.8	88.5
**Intense stare ≥ 5s**	**40.0**	**55.3**	**76.9**
**Repeated tail swishing not in synchrony with spur aids**	**37.0**	**52.6**	**80.8**
**Ears back behind vertical ≥ 5s**	**19.0**	**39.5**	**53.9**
**Repeated head tilt**	**56.0**	**10.5**	**30.8**
**Spontaneous change of gait**	**2.5**	**15.8**	**19.2**
**Repeated stumbling or bilateral hindlimb toe drag**	**0**	**26.3**	**34.6**
**Repeated exposure of the sclera**	**22.0**	**7.9**	**11.5**
**Repeated exposure of the tongue**	**8.0**	**18.4**	**19.2**
**Head moved from side to side**	**10.0**	**5.3**	**3.9**
Spontaneous change of direction; spooking	2.5	2.6	0
Head movement up and down, not in synchrony with the	5.0	5.3	0

trot rhythm			
Bucking	2.5	2.6	3.9
Rearing	0	5.3	0
Reluctance to go forwards	1.5	7.9	3.9
Crooked, on 3 tracks	4.5	0	7.7
Crooked tail, held to one side	26.0	13.2	23.1
Eyes partially closed 2-5s; repeated blinking	0	0	0
Gait too slow	1.5	0	0
Rushed gait	0	0	0
**Repeated incorrect strike off in canter**	**2.0**	**0**	**0**
**Bit pulled through to one side**	**4.5**	**0**	**0**
Head in front of vertical $\geq 30^0 \geq 10$s	0	0	0
